# Oncological aspects related to the non-surgical treatment of basal cell carcinoma – Response^⋆^

**DOI:** 10.1016/j.abd.2023.11.002

**Published:** 2024-02-02

**Authors:** Paulo Rodrigo Pacola

**Affiliations:** Department of Dermatology, Hospital Universitário Júlio Muller, Universidade Federal de Mato Grosso, Cuiabá, MT, Brazil

*Dear Editor,*

The authors are grateful for the comments regarding the article: Chemotherapeutical treatment of basal cell carcinoma with bleomycin via microinfusion of the drug into the skin (MMP®)^1^ and are aware of the main limitation of the study, which was the six-month follow-up. These controls were carried out after six months, through spindle biopsies and histopathology, which is the standard method for diagnosis.^2^ It is also known that only micrographic surgery using the Munich technique could assertively confirm the absence of disease throughout the scar, even five years after the treatment.^3^

Although the study ended in 2021, the patients continue to be monitored, so that after five years this cure rate can be updated and a new report can be published.

Regarding safety margins, it is our knowledge that low-risk lesions require at least 4 mm and high-risk lesions require at least 6‒7 mm,^4^, ^5^ and these guidelines were followed for the treated lesions.

Regarding tumor thickness, as mentioned before, few lesions with tumor thickness >3 mm were included. In fact, only one of them showed recurrence within six months. In this case, this decision was made because it was located in a low-risk area and was elevated above skin level. This observation is necessary to recall that it was a criterion of the study to perform a shave biopsy in all lesions that were elevated above the normal skin surface (safety margin region) so that the penetrating needle reached the same depth both in the lesion and in the safety margin. And once the lesion is “leveled”, the tumor thickness decreases.

Considering the low risk of metastasis of basal cell carcinoma and the greater risk of local recurrence, it was decided to include such lesions, as well as lesions in high-risk areas so that there was a broader view of the therapeutic potential of the bleomycin technique in this study.

The high rate (19%) of patients lost to follow-up was due to factors beyond the authors knowledge. And, in principle, not associated with any type of complication, so it cannot be stated or concluded that it had any relationship with the initial treatment and may be the result of sociocultural characteristics of the attended population. Therefore, these data were excluded from the analyses. It is important to emphasize that all patients were advised to return for follow-up. And, according to the commitment established through the FIC form, the post-surgical follow-up during five years was our responsibility, as well as offering other treatment options in case of recurrence.

We are thankful for the considerations, as they are always important and relevant for our improvement.

## Financial support

None declared.

## Authors' contributions

Paulo Rodrigo Pacola: Approval of the final version of the manuscript; design and planning of the study; drafting and editing of the manuscript.

## Conflicts of interest

None declared.
